# Behavioral Variant Frontotemporal Dementia as a Catalyst for Caregiver Stress Syndrome: A Neuropsychiatric and Sociopolitical Examination

**DOI:** 10.1192/j.eurpsy.2025.1635

**Published:** 2025-08-26

**Authors:** V. H. Santos, I. A. Silva, C. Oom, R. P. Andrade, Z. C. Esá, B. Sousa, T. Carvalhão, S. Fontes

**Affiliations:** 1 Department of Psychiatry and Mental Health, Cova da Beira Local Health Unit; 2 Faculty of Health Sciences, University of Beira Interior, Covilhã; 3 Department of Psychiatry and Mental Health, Norte Alentejano Local Health Unit, Portalegre; 4 Department of Psychiatry and Mental Health, Estuário do Tejo Local Health Unit, Vila Franca de Xira; 5 Department of Psychiatry and Mental Health, Viseu Dão-Lafões Local Health Unit, Viseu, Portugal

## Abstract

**Introduction:**

Behavioral variant frontotemporal dementia (bvFTD), characterized by profound personality changes, impulsivity, and disinhibition, is a neurodegenerative disorder that imposes a significant burden on caregivers. This condition is increasingly recognized as a standalone factor contributing to the onset of caregiver stress syndrome (CSS). This case explores the intricate interplay between bvFTD’s neurobehavioral manifestations, its effect on caregivers, and the systemic insufficiencies in offering support.

**Objectives:**

To highlight the correlation between behavioral variant frontotemporal dementia (bvFTD) and caregiver stress syndrome, emphasizing the systemic limitations in social and financial aid for caregivers.

**Methods:**

A 68-year-old female presented with significant behavioral alterations, including disinhibition and impulsivity. These manifestations, coupled with frontal and temporal lobe atrophy confirmed via CT scan, pointed to a diagnosis of bvFTD. Her daughter, the primary caregiver, developed significant psychological distress in response to the escalating caregiving demands. The daughter’s request for caregiver status, and subsequent efforts to obtain financial and psychosocial support, were met with significant systemic barriers.

**Results:**

In line with broader data, caregivers of individuals with dementia—especially bvFTD—exhibit disproportionately high levels of psychological distress. The daughter’s subsequent psychological symptoms mirrored national trends of caregiver overload, exacerbated by insufficient systemic response and support. Studies show that caregiver burden tends to increase over time, with upward stress trajectories due to increased caregiving demands and reduced social support. This burden can precipitate mental health problems, such as mood disorders, and increase caregiver mortality risk significantly.

**Conclusions:**

The behavioral disturbances characteristic of bvFTD intensify the psychological burden on caregivers, who must contend with the rapid cognitive and functional decline of their loved ones. The absence of sustained financial and psychosocial support exacerbates this strain, pointing to a critical need for reforms in caregiving policies. Understanding the clinical trajectory of bvFTD and its unique caregiver challenges is paramount for developing targeted interventions to prevent caregiver burnout, improve patient outcomes, and mitigate the psychosocial and health risks for caregivers.

**Disclosure of Interest:**

None Declared

